# Propofol Sedation in Pediatric Upper Endoscopy: A Study of Pharmacodynamics and the Effects of Gastroenterologists, Anesthesiologists, and Supervised Participants on the Procedure Time and Sedation Time

**DOI:** 10.7759/cureus.54841

**Published:** 2024-02-24

**Authors:** Ahila Manivannan, Shailender Madani, Michael Woodall, George McKelvey, Sharon Kemper

**Affiliations:** 1 Internal Medicine, Henry Ford Health, Detroit, USA; 2 Pediatric Gastroenterology, Children's Hospital of Michigan, Troy, USA; 3 Pediatrics, NorthShore/Endeavor Health Medical Group, Evanston, USA; 4 Anesthesiology, Detroit Medical Center, Detroit, USA; 5 Pediatric Anesthesiology, Children's Hospital of Michigan, Detroit, USA

**Keywords:** pediatric anesthesiology, sedation, bmi, pediatric gastroenterology, propofol

## Abstract

Background and aims

Propofol combined with fentanyl is a commonly used sedative for pediatric upper endoscopies (UEs). The primary aim was to study the association between propofol dose and procedure and sedation time. The secondary aims were to assess the pharmacodynamics of propofol use with fentanyl and evaluate if gastroenterologists’ and anesthesiologists’ years of experience or the presence of supervised participants (such as students, residents, and fellows) have any influence on the procedure and sedation time.

Methods

A retrospective study was performed at the Children’s Hospital of Michigan on patients under 18 years who underwent UEs with propofol sedation with fentanyl over a two-year period.

Results

A correlation was found between the propofol amount used expressed per body mass index (BMI)/body surface area (BSA), procedure time, and sedation time (p < 0.0001). Throat pain was the most common post-procedural adverse event (4.48%). The impact of psychoactive drugs on these events was not statistically significant, but attention-deficit/hyperactivity disorder (ADHD) medication use was related to increased post-procedural pain complaints. The use of prescribed psychoactive medications was associated with larger propofol dose usage (p = 0.007) without a significant increase in sedation time. Individual gastroenterologists, their years of experience, and the presence of supervised participants were associated with different procedure times (p <0.0001, <0.0001, 0.01). Fellow participation was associated with a 1.11-minute procedure time increase (p = 0.04). Individual anesthesiologists, their years of experience, and the presence of supervised participants were associated with different sedation times (p <0.0001, <0.0001, 0.01).

Conclusion

We found a novel correlation between propofol dosing expressed by the BMI/BSA and sedation time. The UE procedure time and sedation time are associated with individual gastroenterologists and anesthesiologists, their years of experience, and the presence of supervised participants.

## Introduction

Unlike in adults, upper endoscopies (UEs) in children are often performed under deep sedation. There is currently not a standard sedation regimen for the drugs currently available, which include propofol, midazolam, ketamine, and fentanyl, individually or their combination [1]. At present, propofol is the most commonly used agent by anesthesiologists in hospital settings for UEs in children. It is seen to be both safe and convenient, with a rapid onset of action [2]. Propofol is a sedative-hypnotic that is highly lipophilic, which allows it to cross the blood-brain barrier rapidly and act quickly. The depth of sedation is dose-dependent, and it has a narrow therapeutic index [3]. Propofol is typically administered as a loading bolus of 1 mg/kg of body weight and titrated to achieve the appropriate level of deep sedation, which is when the patient cannot be easily aroused but will respond to repeated or painful stimuli [4].

Propofol is commonly combined with fentanyl for several reasons. In a meta-analysis comparing propofol-only regimes versus other sedation regimens in pediatric endoscopies, there was a significantly higher incidence of cardiopulmonary complications in the propofol-only group [5]. When used singly, propofol must be used in relatively large doses to achieve an adequate level of sedation. These large doses can lead to serious side effects, such as hypotension or respiratory depression, with no reversal agent. A smaller dose of propofol is needed when combined with fentanyl [6], as studies have shown that fentanyl decreases propofol requirements in a synergistic manner [7]. Adding fentanyl is also beneficial due to its analgesic properties, a feature that propofol lacks. Endoscopies done with only propofol can result in airway stimulation and gagging, which can cause early termination of the procedure [8].

The overall rate of complications during pediatric UEs is 2.3%, and complications are most associated with younger patients, higher American Society of Anesthesiologists (ASA) class, female gender, and intravenous sedation use [9]. The procedure itself is safe, with sore throat and hoarseness as the most common adverse events, with bleeding and perforation as rare occurrences [10].

Although one would expect increased efficiency with longer clinical practice, little is known about the years of experience of gastroenterologists after pediatric gastroenterology fellowship training and its effect on procedure time. Similarly, little is known about the years of experience of anesthesiologists after pediatric anesthesia fellowship and its effect on sedation time. There is also a paucity of evidence regarding the effects of participants and trainees on UE procedure time and sedation time.

Conventionally, propofol is dosed as mg/kg [4]. At our institution, we have seen patients requiring a wide range of propofol dosing beyond the initial propofol bolus for maintaining an adequate level of sedation for the safe accomplishment of UEs. We designed this study to determine the effect of propofol dose (in terms of total dose, mg/kg, body mass index (BMI), and body surface area (BSA)) on the UE procedure time and sedation time and to study the impact of primary participants (gastroenterologists and anesthesiologists), with and without supervised participants (SPs).

## Materials and methods

Aims

The primary aim was to study the association among the propofol dose, procedure time, and sedation time. The secondary aims of the study were to assess the pharmacodynamics of propofol use with fentanyl in pediatric UEs and to evaluate if the gastroenterologists’ and anesthesiologists’ years of experience or presence of SPs have any influence on the procedure time or sedation time, respectively.

Methods

This was a retrospective study performed at the Children’s Hospital of Michigan as approved by the institutional review board (012616MP4E, dated January 26, 2016). This study consisted of a chart review of all children 18 years and under who underwent diagnostic UE alone, with ASA categories I-III, performed by seven pediatric gastroenterologists, under propofol anesthesia with fentanyl administered by 20 pediatric anesthesiologists at the Children’s Hospital of Michigan during a two-year period from 2014 to 2015. Those patients who had multiple procedures (i.e., UE and colonoscopy) while under the same procedural sedation were excluded from the review. The patients in this study underwent UE in the anesthesia procedure unit (APU), a low-acuity setting that is similar to an ambulatory endoscopy suite. As in any hospital/free-standing procedural unit, there is typically a primary gastroenterologist and anesthesiologist on record responsible for the performance of the UE and inducing sedation, respectively. Like many teaching hospitals, ours includes fellows, residents, medical students, certified registered nurse anesthesiologists (CRNAs), and CRNA students who participate under the supervision of the primary participants. For the purpose of this study, we are identifying them as SPs.

All patients in this study were sedated by a pediatric anesthesiologist. The standard induction utilized by our anesthesiologists started with an inhalation induction with 70% nitrous oxide and 30% oxygen, with the inhalant sevoflurane increased quickly up to 8%. A bolus of 0.0005-0.001 mg/kg of fentanyl was administered. This was followed by 1 mg/kg of propofol, with a continuous infusion of propofol between 0.25 and 0.3 mg/kg/min. Oxygen saturation, blood pressure, heart rate, and level of sedation were monitored. The infusion rate and dose of propofol were adjusted according to the oxygenation and ventilation status and the level of sedation. To offset nausea and vomiting that can sometimes occur with sedation and anesthesia, dexamethasone and ondansetron were given. The appropriate level of sedation was generally determined to be the absence of gagging or coughing during the insertion of the endoscope. The pediatric gastroenterologist performed the UE in a standard manner, with the endoscopy process being initiated once the child was adequately sedated.

Data collection included the details of age, gender, psychiatric and neurologic medications reportedly taken in the two weeks prior to enrollment, and the indication for UE. Procedure time was considered as the difference between the time of insertion of the endoscope and its removal, as per nursing documentation. Sedation time was calculated as the difference between the time of induction and the time the patient exited the unit, as per the nursing documentation. Intra-procedural adverse events as detailed by the gastroenterologist and anesthesiologists were extracted. Post-procedural adverse events as reported by the patients on routine phone calls made by the designated follow-up nurses the following day were extracted. Cases were grouped according to the operating gastroenterologist to study the role of years of experience in the time taken to perform the procedure. Gastroenterologists were grouped according to when they graduated from fellowship using 2003 as the dividing year, as it was the median year of graduation. As with the gastroenterologists, the time taken by the anesthesiologists to perform the sedation was examined in relation to years of experience. Anesthesiologists were grouped according to when they graduated from fellowship using 1997 as the dividing year, as it was the median year of graduation. Total doses of propofol were recorded and calculated per kilogram (kg), per BMI, and per BSA. The use of other medications provided during the procedure were also captured. 

All statistical procedures were performed with IBM SPSS Statistics for Windows, version 20 (released 2011; IBM Corp., Armonk, New York, United States). Statistical significance was considered achieved at a p-value <0.05, two-tailed. Where applicable, differences in proportions between selected variables were compared using a Fisher’s exact chi-square test. Where applicable, differences in mean values were examined up to two decimal places using either parametric independent sample t-tests, non-parametric Mann-Whitney U tests, or non-parametric Kruskal-Wallis tests. When applicable, Bonferroni/Dunn post-hoc analysis was used.

## Results

Of the 1,777 charts reviewed, 1,323 had appropriately documented propofol doses, fentanyl doses, height, and weight. Population demographics are detailed in Table 1, and the propofol dosing in relation to procedure and sedation time are detailed in Table 2. We noted a strong association between increased propofol dosing and longer procedure and sedation times (p < 0.0001). The mean fentanyl dose used was 35. 04 ± 20.77 mg.

**Table 1:** Population demographics * Other preoperative diagnoses include failure to thrive, feeding difficulties, diarrhea, constipation, and weight loss. ** Other medications include amitriptyline, gabapentin, and clonidine. BMI: body mass index, ASA: American Society of Anesthesiologists, GERD: gastroesophageal reflux disease, ADHD: attention-deficit/hyperactivity disorder

Characteristics	n	Percentage
Gender		
Male	697	53%
Female	626	47%
Age		
< 5 years old	65	5%
5-9 years old	407	31%
10-14 years old	530	40%
>15 years old	321	24%
BMI		
<18.5	590	45%
18.5-24.9	480	36%
25-29.9	149	11%
>30	104	8%
BSA		
<0.5	1	0.1%
0.5-0.9	208	15.7%
1-1.4	507	38.3%
1.5-1.9	494	37.3%
2-2.4	99	7.5%
2.5-2.9	14	1.1%
ASA category		
1	330	25%
2	899	68%
3	94	7%
Preoperative diagnosis		
Abdominal pain	559	42%
GERD	259	20%
Eosinophilic esophagitis	211	16%
Vomiting	95	7%
Dysphagia	79	6%
*Other	71	5%
Celiac disease	24	2%
Helicobacter pylori	25	2%
Patients on prescribed psychoactive drugs		
None	1175	89%
ADHD medications	58	4%
Antidepressant medications	35	3%
Antiepileptic medications	26	2%
**Other medications	17	1%
Antipsychotic medications	12	1%

**Table 2:** Association of propofol dosing with the procedure time and sedation time * statistical significance BMI: body mass index, BSA: body surface area

		Propofol dose relationship to the procedure time	Propofol dose relationship to the sedation time
Propofol dose	Mean ± SD (mg)	p-value	p-value
Expressed as the total dose (mg)	144.06 ± 93.87	<0.0001*	<0.0001*
Expressed as the dose per weight (mg/kg)	3.50 ± 2.64	0.003*	0.0064*
Expressed as the dose per BMI (mg/kg/m^2^)	7.06 ± 4.39	<0.0001*	<0.0001*
Expressed as the dose per BSA (mg/m^2^)	109.18 ± 70.62	<0.0001*	<0.0001*

Details of the dosing of propofol during UE per weight, BMI, and BSA are detailed in Table 3. Post-procedural adverse events are noted in Table 4. The only two gastroenterological intra-procedural adverse events noted were a mucosal tear at the mid esophagus at the site of a stricture in one patient and a submucosal hematoma in the duodenum after biopsy in another. There were no reported sedation-related intra-procedural adverse events. Regarding immediate post-procedural sedation-related adverse events, 11 patients exhibited gagging, four patients reported nausea, and five patients vomited. Observed frequencies for post-procedural adverse events reported on follow-up phone calls were 17.28% (14/81) in patients who were prescribed psychoactive drugs and 17.31% (102/589) in patients who were not. The chi-square p-value for the two groups is 0.99; thus, the impact of psychoactive drugs on reported post-procedural adverse events was not statistically significant. Of note, 631 patients were not able to be reached for a follow-up phone call. A percentage (21.2%) of patients prescribed ADHD medications had reported adverse events, all regarding either generalized pain or pain in the throat or abdomen, while 17.1% of the patients not prescribed ADHD medications had reported adverse events. The chi-square p-value for the two groups was 0.6; thus, the impact of the prescribed ADHD medications on post-procedural events was not statistically significant. However, the use of prescribed psychoactive medications was associated with a larger propofol dose usage (p-value of 0.007) without a significant increase in sedation time (p-value of 0.2). Such patients were given an average of 21.98 mg more propofol than patients that were not prescribed psychoactive medications, with an associated non-significant 1.04-minute increase in sedation time.

**Table 3:** Propofol dosing in upper endoscopy UE: upper endoscopy, BMI: body mass index, BSA: body surface area, SD: standard deviation

Dosing of propofol to complete UE	Mean ± SD
Expressed as the total dose (mg)	144.06 ± 93.87
Expressed as the dose per weight (mg/kg)	3.50 ± 2.64
Expressed as the dose per BMI (mg/kg/m^2^)	7.06 ± 4.39
Expressed as the dose per BSA (mg/m^2^)	109.18 ± 70.62

**Table 4:** Post-procedural events

Post-procedural adverse events	n	Percentage
None	554	80.06%
Throat pain/sore throat	31	4.48%
Unspecified pain	27	3.90%
Abdominal pain	18	2.60%
Nausea	11	1.59%
Vomiting	10	1.45%
Chest pain	9	1.30%
Cough	8	1.16%
Fever	8	1.16%
Headache	7	1.01%
Refusing oral intake	2	0.29%
Diarrhea	1	0.14%
Eye pain	1	0.14%
Lightheadedness	1	0.14%
Numb tongue	1	0.14%
Numbness in arms and hands	1	0.14%
Vomited small amount of blood	1	0.14%
Rash	1	0.14%

Tables 5-10 detail the effect that gastroenterologists, anesthesiologists, and their SPs had on the procedure and sedation time. Individual gastroenterologists were associated with different procedure times (p < 0.0001) (Table 5). Gastroenterologists who graduated fellowship after 2003 were associated with longer procedure times than those who graduated fellowship before 2003 (p < 0.0001) (Table 6). Individual anesthesiologists were associated with different sedation times (p < 0.0001) (Table 7). Anesthesiologists who graduated fellowship after 1997 were associated with longer sedation times than those who graduated fellowship before 1997 (p < 0.0001) (Table 8). As seen in Table 9, there were significant differences in procedure time among gastroenterologists' SPs (p = 0.01). Fellow participation was associated with a statistically significant 1.11-minute increase in procedure time (p = 0.04). As seen in Table 10, there were also significant differences in the sedation time among anesthesiologists' SPs (p = 0.01). There were no significant differences in adjusted comparisons between anesthesiology participant groups (p > 0.05). We have discounted the statistical significance of medical students being associated with shorter sedation times compared to fellows because medical students do not perform the procedure.

**Table 5:** Individual gastroenterologists and their procedure times

Gastroenterologist	n	Mean time ± SD (min)
GA	255	5.77 ± 5.10
GB	17	6.94 ± 2.73
GC	300	6.98 ± 3.33
GD	227	7.68 ± 5.97
GE	293	7.00 ± 7.42
GF	139	10.40 ± 5.71
GG	92	7.74 ± 2.10

**Table 6:** Gastroenterologists’ experience and impact on the procedure time

	n	Procedure time ± SD (min)
Gastroenterologists who graduated fellowship before 2003	383	6.73 ± 5.34
Gastroenterologists who graduated fellowship after 2003	940	8.64 ± 5.92

**Table 7:** Individual anesthesiologists and their sedation times

Anesthesiologist	n	Mean time ± SD (min)
AA	37	22.27 ± 4.61
AB	288	20.43 ± 6.10
AC	60	25.33 ± 7.13
AD	115	22.17 ± 6.44
AE	6	17.83 ± 3.19
AF	20	26.65 ± 6.84
AG	118	23.04 ± 11.04
AH	68	21.68 ± 6.62
AI	25	27.52 ± 18.86
AJ	127	23.32 ± 7.20
AK	6	19.00 ± 4.10
AL	22	29.86 ± 11.05
AM	8	27.50 ± 5.10
AN	88	25.71 ± 13.19
AO	88	22.00 ± 6.41
AP	31	25.39 ± 12.51
AQ	17	25.18 ± 10.35
AR	163	22.09 ± 6.46
AS	6	33.83 ± 14.15
AT	30	23.27 ± 5.07

**Table 8:** Anesthesiologists’ experience and impact on the sedation time

	n	Sedation time ± SD (min)
Anesthesiologists who graduated fellowship before 1997	867	21.97 ± 7.38
Anesthesiologists who graduated fellowship after 1997	456	24.38 ± 10.09

**Table 9:** Gastroenterologists’ supervised participants and their association with the procedure time

Participant	n	Mean time ± SD (min)
None	964	7.17 ± 5.81
Medical student	32	5.94 ± 2.24
Pediatric resident	199	7.56 ± 5.56
Gastroenterology fellow	98	8.28 ± 4.50
Other	30	7.30 ± 3.02

**Table 10:** Anesthesiologists’ supervised participants and their association with the sedation time

Participant	n	Mean time ± SD (min)
None	109	22.89 ± 8.49
Certified registered nurse anesthetist	620	22.54 ± 8.49
Certified registered nurse anesthetist student	137	24.50 ± 8.98
Anesthesia resident	349	22.05 ± 7.82
Pediatric anesthesia fellow	46	24.85 ± 7.45
Other	62	24.19 ± 10.90

## Discussion

Our study found a strong association between increased propofol dosing and longer procedure and sedation times. It stands to reason that the depth of sedation using propofol is dose-dependent [3]. Longer procedure times likely in turn required more propofol to maintain sedation. One would expect that propofol dosing, whether expressed per BMI/BSA or mg/kg, would be statistically similar when related to the procedure time and sedation time. However, our study’s finding of a stronger statistical correlation of propofol dosing per BMI and BSA to the procedure time and sedation time compared to mg/kg is a provocative distinction without a readily apparent cause. The dosing of propofol is subjective and can lead to a wide range of dosing, which in turn can lead to an increased risk of cardiopulmonary events and respiratory depression. Our study’s propofol dosing falls within the dosing range reported in the literature with regard to mg/kg dosing [11,12]. Even though the correlation to the procedure time and sedation time is not dramatically different between the different propofol dosing, the BMI and BSA should be considered as methods of calculating propofol dose, especially in dosing underweight and overweight children, where per kg dosing may be less ideal. Interestingly, one adult study that used propofol found that the UE time was 0.21 minutes longer for every additional BMI unit, which was similar to our study’s 0.34 minutes, suggesting that the BMI can potentially be used as a predictor of procedure time [13]. This must be investigated further in prospective studies to consider the viability of creating a standardized dosing regimen for propofol with fentanyl based on BMI or BSA for pediatric UEs, focusing on the safety of that sedation regime. Our study shows that the dosing of propofol based on the BMI and BSA for pediatric UEs is a valid method and safe when used in an ambulatory procedure center setting.

Regarding the safety profile of propofol use in combination with fentanyl, we have shown that this combination is associated with minimal gastroenterological and sedation-related intra-procedural adverse events, with no serious cardiopulmonary events recorded. Iatrogenic adverse events from UEs are rare, occurring with an incidence of 2.3% in pediatric patients [14], which is in line with our study’s reported 1.7%. While the intra-procedural safety of propofol use with fentanyl in pediatric UEs has been studied [15,16], few have examined the symptoms or adverse events reported after the procedure. One study that focused on pediatric UEs reported that throat pain was the most common adverse event in children after UE [17], which is congruent with our findings. A prospective observational study found that 2.6% (249/9577) of pediatric UEs were associated with a post-procedural adverse event, which is much lower than our reported 20% incidence. However, 160 of their patients had to seek medical care while none of our patients did, which suggests that the acuity of our patients’ adverse events was lower [18]. This could also be related to the difference in the nature of the studies, i.e., prospective versus retrospective. A standardized post-procedural surveillance system should be implemented, and more studies should compare the adverse events reported after UE with the various sedation protocols currently being utilized.

Premorbid conditions and medication usage are known to impact anesthesia requirements, but little is known about their impact on adverse events following UE. Our study finding of an increased propofol dose needed for patients on psychoactive medications without a significant increase in sedation time cannot be readily explained. Previous studies suggest that certain antiepileptic medications inhibit propofol metabolism, which decreases the propofol dose requirement. However, these relationships are not fully understood [19,20]. As the number of patients in each category of medications was small, we considered them as one group, although the mechanisms of these medications are different. Any meaningful analysis between these groups would have to be done with larger groups in prospective studies. Our results are underpowered to examine a correlation between patients on specific psychoactive medications and adverse events other than with the patients prescribed ADHD medications because only half of our patients responded to follow-up phone calls. Previous studies have been unable to discern any influence of ADHD on propofol requirements, except for these patients being less cooperative with the initiation of anesthesia [21]. Our study found that while there were not significantly more post-procedural adverse events in children prescribed ADHD medications, they did have an increased rate of pain complaints after the procedure. Possible considerations include that these medications alter the metabolism of the medications provided during the procedure, that patients with ADHD have an altered sensation of pain, and that these patients are more likely to verbalize discomfort compared to other patients. We are limited in discerning this relationship by not knowing if that pain was pre-existing, new, or worsened from before/after the procedure. Detailing pre- and post- procedure symptoms would be useful to distinguish side effects and continuing complaints. Further research is needed to better understand the causality of these relationships.

While there are reports detailing the relationship between a surgeon’s years of experience and surgery time [22], none are focused on gastroenterologists. Our finding of an association between the individual gastroenterologist performing the UE and their years of experience with procedure time is a novel observation not reported in the literature. This finding is likely the result of years of experience improving time efficiency and skill with which the UE can be performed. Further studies that examine how many years in practice do newer attendings decrease procedure time may help in determining the optimal procedure unit scheduling time.

Gastroenterologists are slowed by having SPs, specifically fellows, because they were guiding the fellows through the procedure. In Mark and Kramer's study, there was a 36.4% fellow involvement in pediatric UEs [23], compared to 7% in ours. One explanation for this disparity is that our fellows mostly attended procedures done in the operating room rather than the APU. It was not clearly stated in their paper whether the procedures were performed in the operating room versus the procedure unit. Congruent to our study findings, they also found a significant difference in the procedure time with fellow involvement versus attending-alone procedures [23]. Fellows are the most likely SPs to increase procedure time as they are the most involved in the procedure while trying to learn proper techniques. Although the increase in time is minimal, on average 1.11 minutes, awareness of this increase may help in scheduling cases appropriately and adjusting expectations.

Just like how gastroenterologists’ efficiency improved with time, so did anesthesiologists’. There are currently no studies that focus on the relationship between anesthesiologists and sedation time in UEs. It is likely that with years of experience, anesthesiologists become more skillful in managing the patient’s level of sedation. The only study that examined individual anesthesiologists’ effect on sedation time was in the surgical setting, which found their impact to be negligible [22].

Our study found the presence of SPs with the anesthesiologist was associated with an increased sedation time, but the time increase was relatively small, with the largest average time difference being 2.31 minutes. This added time could be the result of allowing learners to place an IV and learn protective airway management techniques, both of which become streamlined later in practice. Increased sedation times could also have a cost impact. The use of anesthesiologist-administered propofol for low-risk endoscopies has been estimated to cost the United States healthcare system an additional $3.2 billion USD over a span of 10 years [24]. Eappen et al. found that there was a statistically significant increase in time for induction and emergence from anesthesia in the operating room when done by new anesthesia residents compared to attendings working alone [25]. However, they found that the increase in time did not correlate to a meaningful effect on cost or adverse events. As such, studies are not present for APUs to our knowledge, it would be interesting to see if this association extends beyond the operating room. Moreover, as avoiding prolonged sedation in pediatric patients is ideal, factors that increase sedation time should be studied more closely.

The strengths of our study were that it was carried out in a large cohort at a tertiary care center. Our study focused on gaps in knowledge in the use of propofol with fentanyl in pediatric UEs, which is a field that is less researched than that of the adult world. According to our institutional mandates, the propofol dosing, procedure time, and sedation time were entered in real time and can be relied upon.

Our study has limitations inherent to a retrospective study. We were limited in that only half of our patients responded to the follow-up phone call. Thus, information bias in parental reporting of post-procedural adverse events is possible. Due to the small number of patients prescribed psychoactive medications, we were not able to examine the effects of these medications on propofol dosing closely.

## Conclusions

Our study found an association between the propofol dose and the procedure time and sedation time and detailed the pharmacodynamics of propofol use with fentanyl in pediatric UEs. We have found a previously unreported increased correlation between propofol dosing expressed by the BMI/BSA, rather than by per mg/kg, and the procedure time and sedation time. The UE procedure time and sedation time are associated with individual gastroenterologists, anesthesiologists, their years of experience, and the presence of SPs. These findings should be confirmed in prospective multicenter studies.
